# High-Yield Production of Catalytically Active Regulatory [NiFe]-Hydrogenase From *Cupriavidus necator* in *Escherichia coli*

**DOI:** 10.3389/fmicb.2022.894375

**Published:** 2022-04-29

**Authors:** Qin Fan, Giorgio Caserta, Christian Lorent, Ingo Zebger, Peter Neubauer, Oliver Lenz, Matthias Gimpel

**Affiliations:** ^1^Chair of Bioprocess Engineering, Department of Biotechnology, Technische Universität Berlin, Berlin, Germany; ^2^Department of Chemistry, Technische Universität Berlin, Berlin, Germany

**Keywords:** regulatory hydrogenase, difficult-to-express protein, *Escherichia coli*, [NiFe]-hydrogenase, *Cupriavidus necator*

## Abstract

Hydrogenases are biotechnologically relevant metalloenzymes that catalyze the reversible conversion of molecular hydrogen into protons and electrons. The O_2_-tolerant [NiFe]-hydrogenases from *Cupriavidus necator* (formerly *Ralstonia eutropha*) are of particular interest as they maintain catalysis even in the presence of molecular oxygen. However, to meet the demands of biotechnological applications and scientific research, a heterologous production strategy is required to overcome the low production yields in their native host. We have previously used the regulatory hydrogenase (RH) from *C. necator* as a model for the development of such a heterologous hydrogenase production process in *E. coli*. Although high protein yields were obtained, the purified enzyme was inactive due to the lack of the catalytic center, which contains an inorganic nickel-iron cofactor. In the present study, we significantly improved the production process to obtain catalytically active RH. We optimized important factors such as O_2_ content, metal availability, production temperature and time as well as the co-expression of RH-specific maturase genes. The RH was successfully matured during aerobic cultivation of *E. coli* by co-production of seven hydrogenase-specific maturases and a nickel permease, which was confirmed by activity measurements and spectroscopic investigations of the purified enzyme. The improved production conditions resulted in a high yield of about 80 mg L^–1^ of catalytically active RH and an up to 160-fold space-time yield in *E. coli* compared to that in the native host *C. necator* [<0.1 U (L d) ^–1^]. Our strategy has important implications for the use of *E. coli* K-12 and B strains in the recombinant production of complex metalloenzymes, and provides a blueprint for the production of catalytically active [NiFe]-hydrogenases in biotechnologically relevant quantities.

## Introduction

[NiFe]-hydrogenases are metalloenzymes that catalyze the reversible oxidation of molecular hydrogen (H_2_) into protons and electrons, which makes them very attractive from both scientific and applied perspectives (Fontecilla-Camps et al., 2007; Lubitz et al., 2014). Their core module is composed of a large subunit harboring the catalytic nickel-iron [NiFe] center and a small subunit hosting one to three iron-sulfur clusters that mediates electron transfer between the active site and the physiological electron acceptor/donor (Ogata et al., 2016). The inorganic [NiFe] center is coordinated to the protein *via* four cysteine-derived thiolates, two of which are terminal nickel ligands whereas the other two serve as bridging ligands between the nickel and the iron. The iron is further coordinated by one carbonyl (CO) group and two cyanide (CN^–^) residues (Fontecilla-Camps et al., 2007; Lubitz et al., 2014; Peters et al., 2015). Synthesis and incorporation of the [NiFe] cofactor into the apo-hydrogenase is a highly complex process that requires a set of at least six maturation proteins, named HypABCDEF (Forzi and Sawers, 2007; Lacasse and Zamble, 2016). The HypCD complex serves as central scaffold for the assembly of the Fe(CN)_2_CO moiety (Blokesch et al., 2004a; Bürstel et al., 2012; Watanabe et al., 2012). The cyanide ligands are synthesized from carbamoyl phosphate by a concerted action of HypE and HypF (Casalot and Rousset, 2001; Reissmann et al., 2003; Blokesch et al., 2004b; Rangarajan et al., 2008; Lacasse and Zamble, 2016). The source of the CO ligand in anaerobic hydrogenase biosynthesis is still unknown (Bürstel et al., 2011). Among others (Lenz et al., 2007), acetate (Roseboom et al., 2005) and CO_2_ (Soboh et al., 2013) have been suggested as precursors, but compelling evidence is lacking. Under aerobic conditions, however, formyl-tetrahydrofolate serves as the CO precursor (Bürstel et al., 2016). The preformed Fe(CN)_2_CO fragment is then transferred from the HypCD complex into the apo-form of the large hydrogenase subunit (Arlt et al., 2021). Subsequently, the nickel is incorporated by HypA and HypB complex (Lacasse and Zamble, 2016). In *E. coli*, the SlyD protein is also involved in nickel insertion (Leach et al., 2007; Kaluarachchi et al., 2012; Pinske et al., 2015). Upon insertion of the complete [NiFe] cofactor, the hydrogenase large subunit usually undergoes proteolytic cleavage, in which the C-terminal extension is cleaved off (Senger et al., 2017; Pinske et al., 2019; Hartmann et al., 2020). This cleavage step allows oligomerization of the cofactor-containing large subunit with the small subunit, whose Fe-S clusters are incorporated by the universal Isc/Suf machinery.

Due to the complex maturation process, recombinant production of [NiFe]-hydrogenases is challenging, especially in heterologous systems, and makes scalability difficult (Fan et al., 2020). We have chosen the regulatory [NiFe]-hydrogenase (RH) from *Cupriavidus necator* (formerly *Ralstonia eutropha*) as a model for the development of a heterologous hydrogenase production system in *E. coli*. *C. necator* actually possesses four different [NiFe]-hydrogenases, all of which are O_2_-tolerant, i.e., they perform H_2_ conversion even in the presence of molecular oxygen (Fritsch et al., 2013; Schwartz et al., 2013; Lenz et al., 2015). The RH functions as H_2_ sensor in the context of H_2_-dependent transcriptional regulation of the genes encoding the two energy-conserving [NiFe]-hydrogenases of *C. necator* (Lenz et al., 1997, 2010). Like typical [NiFe]-hydrogenases, the RH consists of two subunits, a large subunit HoxC (52 kDa) containing the NiFe(CN)_2_CO center and a small subunit HoxB (36 kDa) harboring three [4Fe-4S] clusters (Pierik et al., 1998; Kleihues et al., 2000). Its H_2_-oxidizing activity is absolutely insensitive to O_2_ (Buhrke et al., 2005a; Ash et al., 2015). The maturation proteins required for cofactor assembly of the RH are encoded by the *hyp1* operon (*hypA1B1F1CDE*) of *C. necator* (Buhrke et al., 2001). In contrast to most other [NiFe]-hydrogenases, the RH large subunit HoxC does not undergo C-terminal processing, making RH a relatively simple [NiFe]-hydrogenase model. Recently, we succeeded to produce the RH heterologously in *E. coli* in a soluble form and purified the protein by a single affinity chromatography step (Fan et al., 2021a). By using an EnPresso B-based fed-batch-like growth mode, we obtained up to 250 mg L^–1^ of RH in shake flask cultures. This RH yield was about 250-fold higher than that from the native producer (Bernhard et al., 2001; Fan et al., 2021a). The productivity has been further improved using IPTG or lactose autoinduction (Fan et al., 2021b). Regrettably, the purified RH turned out to be inactive. While the small subunit appeared to be fully equipped with Fe-S clusters, the large subunit lacked the [NiFe] cofactor, indicating malfunctioning maturation in the heterologous host.

In this study, we aimed at the heterologous production of RH with catalytic activity by testing different *E. coli* strains with various genetic backgrounds and by varying the process conditions. By co-expression of the *hyp1* operon from *C. necator* and the supplementation of the growth medium with NiCl_2_ we obtained RH with catalytic activity. The successful incorporation of the NiFe(CN)_2_CO cofactor was confirmed by IR and EPR spectroscopy. By co-expression of the *hoxN* and *hypX* genes, encoding a nickel permease and a dedicated maturase, respectively, catalytically active RH was also purified from aerobically grown *E. coli* BL21.

## Materials and Methods

### Bacterial Strains, Media and Growth Conditions

While *E. coli* TG1 (Baer et al., 1984) was used for plasmid maintenance, the *E. coli* B strain BL21 Gold (Stratagene, Germany) and the K-12 strains W3110 (Bachmann, 1972) and MC4100 (Casadaban and Cohen, 1979) were used for RH production. Plasmid pQF8 (Fan et al., 2021a) was used for overproduction of structural RH subunits, HoxB_Strep_ and HoxC, under control of the IPTG inducible P_lac–CTU_ promoter. All strains and plasmids are listed in Supplementary Tables 1, 2.

Transformations and plasmid propagations were performed on solid and liquid TY medium (16 g L^–1^ tryptone, 10 g L^–1^ yeast extract, 5 g L^–1^ NaCl, for solid medium 2% agar-agar). The fed-batch-like EnPresso^®^ B medium (EnPresso GmbH, Berlin, Germany) was used for RH production. The EnPresso B medium is based on a typical *E. coli* mineral salt medium supplemented with trace elements and Na_2_SeO_3_, Na_2_MoO_4_ and Ni(NO_3_)_2_ as described previously (Soini et al., 2008). In this medium, glucose is enzymatically released from a non-metabolizable polymer contained therein. For preparation of a pre-culture, *E. coli* cells were inoculated from a single colony in 10 mL LB medium (10 g L^–1^ tryptone, 5 g L^–1^ yeast extract, 10 g L^–1^ NaCl) and shaken for 6–8 h at 37°C, 250 rpm (Infors HT, 25 mm offset, Switzerland). For main cultures, 50 mL EnPresso^®^ B medium was inoculated to an OD_600_ of 0.2 in 250 mL baffled Ultra Yield^®^ shake flask (Thomson Instrument Company, Oceanside, CA, United States) (20% v/v) supplemented with 25 μL of reagent A (1.5 U L^–1^) for overnight cultivation at 30°C, 250 rpm (Infor HT, Switzerland). At the induction point booster and 75 μl reagent A (4.5 U L^–1^) were added according to the manufacturer’s instructions (EnPresso GmbH, Berlin, Germany) and RH production triggered by addition of 50 μM IPTG. The cells were cultivated under the same conditions for 24 h at 30°C or for 48 h/72 h at 18°C. If required all media were supplemented with 25 μg mL^–1^ chloramphenicol and 25 μg mL^–1^ kanamycin for selection.

For induction under O_2_ limited conditions the cultivation protocol was altered as follows. To ensure a high cell density before, a first dose of booster and 75 μl reagent A (4.5 U L^–1^) were added after overnight cultivation and growth continued for 12 h under the same conditions. After 12 h of boosted growth, cultures were transferred into 125 mL baffled PreSens shake flask (40% v/v) which was placed on an SFR shake flask reader (PreSens Precision Sensing GmbH, Regensburg, Germany) in a Kuhner LTX orbital shaker (50 mm offset, Adolf Kühner AG, Basel, Switzerland) with online monitoring of dissolved oxygen (DO) and pH. The oxygen level was controlled by manually reducing the shaking speed from 250 rpm to 100 rpm until a DO value almost close to 0% was reached to ensure O_2_-limited (microaerobic) conditions. A 2nd dose of booster and reagent A was added together with 50 μM IPTG to induce RH production and cultivation continued at 30°C for 36 h or at 18°C for 48 h/66 h/72 h as indicated. Finally, cells were harvested by centrifugation at 8,000 × *g*, 4°C for 10 min. The cell pellets were frozen in liquid nitrogen and stored at −80°C until further use. To investigate an effect of metal ion addition on active RH production, appropriate concentrations of NiCl_2_ or FeSO_4_ were added to the cultures at the induction point and 8 mL boosted cultures were distributed onto the Deep Well OxoDish^®^ OD24-DW deepwell plates with DO online monitoring and cultivated in an SDR SensorDish^®^ Reader (both from PreSens Precision Sensing GmbH, Regensburg, Germany) which was placed in a Duetz plate holder (EnzyScreen BV, Heemstede, Netherlands) and placed and cultivated in the Kuhner LTX shaker (50 mm offset, Adolf Kühner AG, Basel, Switzerland). After 24 h of induction under O_2_-limited cultivation conditions at 30°C cells were harvested from the 8 mL culture suspension as described above.

### RH Purification, Spectroscopic Characterization and Activity Assay

RH purification has been described in detail previously (Fan et al., 2021a,b). All elution fractions were concentrated by ultra-filtration (14,000 × g, 4°C) using Amicon Ultra Ultracel 30 kDa cut-off concentrators (Merk Millipore, Germany). An aliquot of the final concentrate was used for SDS-PAGE. The gels were stained with colloidal Coomassie blue G250 solution and subsequently bands were quantified with ImageJ for determination of protein concentrations. A defined solution of bovine serum albumin served as standard. H_2_-oxidizing activity of RH was measured spectrophotometrically using a Cary50 UV-vis spectrophotometer (Varian, Agilent, Santa Clara, California), and the H_2_ uptake assay using methylene blue as an electron acceptor was used as described previously (Lenz et al., 2018). Measurements were performed with two biological replicates and the standard deviation was calculated from at least two independent technical replicates. For screening experiments of optimal nickel or iron concentrations, the H_2_-oxidation activity was measured in soluble extract of the cell lysates separated from solid cell debris and insoluble fraction after sonication (3 min, 30 s on/off, sonotrode with 3 mm diameter, 30% amplitude) (UP200S, Hielscher GmbH, Germany). The reaction was started by the addition of 200 μl soluble extract to 1.8 mL reaction buffer followed by 100% H_2_ gas saturation as described previously (Fan et al., 2021a). The RH concentration in the soluble extract was quantified in Western blot analysis using known concentration of HoxC as a control. Infrared (IR) and electron paramagnetic resonance (EPR) spectroscopy of RH were measured as described previously (Fan et al., 2021a).

### Construction of Plasmids for *hyp* Gene Expression

Plasmid pGK16, a derivative of the medium copy plasmid pGK14 (Brantl, 1994) carrying a kanamycin resistance gene instead of the erythromycin resistance gene (Gimpel, unpublished) was used as basis for the construction of the *hyp* gene expression plasmids. First, the 267 bp *Sal*I/*Bgl*II fragment from plasmid pGW2 (Schollmeyer, 2020) harboring the P_tac_ promoter, a multiple cloning site and a transcription terminator was cloned into the corresponding pGK14 vector, yielding plasmid pQF11. Next, using Q5 DNA polymerase (New England Biolabs), PCR amplification of the complete *hyp1* operon from pRH-Hyp (Lenz et al., 2007) and *hyp1 (ΔF1)* operon from pRH-Hyp(ΔF1) (Lenz et al., 2007) was performed with oligonucleotides MG0164 (5′-TCATCTAGACGGAGTCTTTGGGAGATACTG-3′) and MG0168 (5′-ACTGCGGCCGCTTAACAAATGCGCGGAAGCT G-3′). The PCR products were digested with *Xba*I and *Not*I and cloned into the pQF11 cut with the same enzymes yielding pQF12 and pQF13. The plasmid pQF12, which contains the complete *hyp1* operon, was used as the basis for the integration of further maturation enzymes. The high-affinity nickel permease encoding *C. necator hoxN* gene was PCR amplified using primers MG0226 (ATCGCGGCCGCACAGGAGACTTCCAGCATGTTCCA) and MG0227 (ACTGCGGCCGCTTAACATGAACTTGTCGGCCAG GA) and plasmid pCH231 (Wolfram et al., 1995) as template. The resulting 0.9 kb fragment was digested with *Not*I and subsequently ligated into the corresponding pQF12 vector yielding plasmid pQF17. The primer pair MG0243 (AGTTCTAGAGCGAGTCGGCTATGCGCATATTGC)/MG024 4 (ATGTCTAGATCAAGATCGTTTCCCCGCAAGTGC) and plasmid pGE771 (Lauterbach and Lenz, 2013) as template were used to PCR-amplify a 1.8 kb fragment encoding the *C. necator* aerobic maturase HypX. The resulting fragment was digested with *Xba*I and ligated into the corresponding pQF17 vector, resulting in plasmid pQF18. The correctness of the amplified sequences was confirmed by sequencing (LGC Genomics, Berlin). A schematic map of all plasmids can be found in Supplementary Figure 1. Expression plasmids were introduced sequentially.

## Results

### Heterologous Production of Catalytically Active RH in O_2_-Limited *E. coli* Cultures

In a previous study we showed that high RH yields can be obtained with *E. coli* BL21 Gold carrying the RH overproduction plasmid pQF8 (strain BQF8RH; Fan et al., 2021a). However, the purified RH did not contain any [NiFe] cofactor (Fan et al., 2021a), which is consistent with major defects of *E. coli* BL21 in metal ion transport and metalloprotein biosynthesis (Pinske et al., 2011). This phenotype has been attributed to a non-sense mutation in the *fnr* gene whose gene product regulates nickel transport and *hyp* gene expression. Global deficiencies in anaerobic metabolism and metal ion transport seem to be common for *E. coli* B derivatives (Pinske et al., 2011). To overcome this obstacle, we transformed the two *E. coli* K-12 derivatives *E. coli* W3110 and *E. coli* MC4100 with plasmid pQF8, resulting into the recombinant strains WQF8RH and MQF8RH, respectively. All strains were cultivated under both aerobic and O_2_-limiting conditions in shake-flasks containing EnPresso B medium, as described in section “Materials and Methods.” O_2_ limitation (DO value close to 0%) was initiated by reducing the shaking speed from 250 to 100 rpm after induction (Supplementary Figure 3). After 24 h of IPTG induction, cells were collected, disrupted, and the RH was purified by affinity chromatography. Furthermore, the H_2_-oxidizing activity of all RH samples was determined.

**FIGURE 1:**
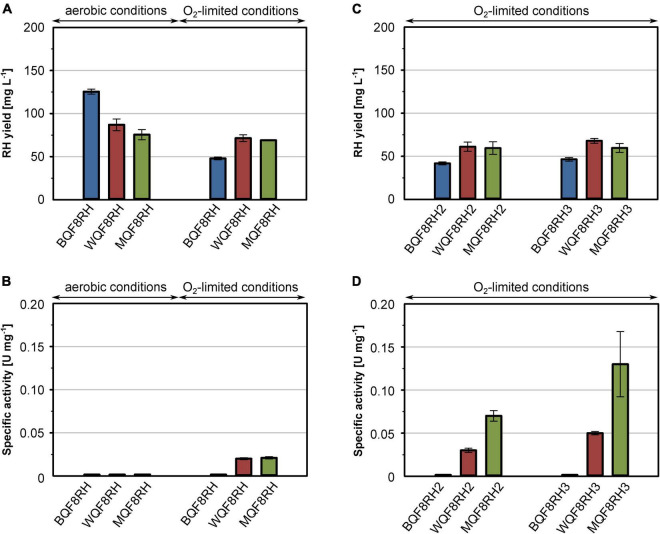
Comparison of production strains, cultivation mode and *hyp* gene co-expression for heterologous RH production. *E. coli* strains BQF8RH, WQF8RH and MQF8RH (derivatives of *E. coli* strains BL21 Gold, W3110 and MC4100, respectively) carry plasmid pQF8 encoding the two RH subunits were used. For co-expression of the maturation genes of *C. necator*, strains BQF8RH, MQF8RH and WQF8RH were transformed with either plasmid pQF12 (encoding the entire *hyp1* operon) or plasmid pQF13 (encoding the modified *hyp1(*Δ*F1)* operon lacking *hypF1*) yielding strains BQF8RH2, WQF8RH2 or MQF8RH2 and BQF8RH3, WQF8RH3 or MQF8RH3, respectively. All strains were cultivated in 50 mL EnPresso B medium as described in section “Materials and Methods.” For aerobic production, cultivation was performed in 250-mL Ultra Yield flasks (20% V/V) shaken at 250 rpm, whereas O_2_-limited production was performed in 125-mL PreSens flasks (40% V/V) adjusted to a DO near 0% by manually decreasing the shaking speed. RH protein was purified by affinity chromatography **(A,C)** and specific activities measured from the purified samples **(B,D)**.

Regardless of the aeration, similar RH protein yields were obtained (data not shown). However, considering the higher final cell densities (Supplementary Figure 2A), the obtained volumetric yields were 1.5–2.5-fold higher under aerobic conditions (Figure 1A). Interestingly, among the three strains tested, BQF8RH performed best under aerobic conditions but worst under O_2_-limiting conditions (Figure 1A and Supplementary Figure 2C), suggesting that anaerobic conditions might be suboptimal for RH production in *E. coli* BL21. Presumably, the lack of certain anaerobic respiration-related proteins and/or enzymes in the B strains prevents rapid adaptation to anaerobic stress (Pinske et al., 2011) and leads to slower growth and lower protein production (Kim et al., 2014). However, the RH purified from BQF8RH showed no activity regardless of the growth conditions (Figure 1B). In contrast, RH preparations from both *E. coli* K-12 derivatives grown under O_2_-limiting conditions exhibited a specific activity of 0.02 U mg^–1^, while RH purified from aerobically grown cells was inactive (Figure 1B). The latter could be due to either the lack of the hydrogenase maturation apparatus or insufficient uptake of nickel ions under aerobic cultivation, both of which are modulated by the transcriptional regulator FNR under anaerobic conditions (Wu et al., 1989; Lutz et al., 1991; Messenger and Green, 2003). Thus, the lack of FNR in *E. coli* B-strains, as well as the reduced FNR levels in the presence of O_2_, might prevent nickel uptake, [NiFe] cofactor assembly, and consequently the synthesis of catalytically active RH. Nevertheless, the RH activities obtained from both *E. coli* K-12 derivatives were quite low and corresponded to only 0.5–1% of those reported for the RH protein isolated from *C. necator*, which is in the range of 1.6–4.5 U mg^–1^ (Buhrke et al., 2005a,b; Caserta et al., 2020). This indicates that the hydrogenase maturation apparatus of *E. coli* K-12 is only able to mature *C. necator* RH to a very limited extent.

### Co-expression of the *C. necator hyp* Genes Improves RH Maturation in *E. coli*

It has been reported previously that co-expression of the *C. necator hyp* genes markedly improve the activity of heterologously produced RH (Lenz et al., 2007). Thus, we constructed plasmids pQF12 and pQF13 carrying the entire *C. necator hyp1* operon [*hypA1B1(F1)CDE*] with or without *hypF1*, respectively, under control of the IPTG-inducible P_tac_ promoter. The plasmids were transferred into strains BQF8RH, WQF8RH, and MQF8RH, resulting in BQF8RH2 and BQF8RH3, WQF8RH2 and WQF8RH3, MQF8RH2 and MQF8RH3, respectively. The strains were cultivated under O_2_-limiting conditions, RH was purified, and its H_2_-oxidation activity was measured. Co-expression of the maturase genes did not significantly affect the RH yield, indicating that the cells tolerate the higher metabolic load (Figures 1A,C). RH purified from strains BQF8RH2 and BQF8RH3 still showed no activity, whereas co-expression of the maturase genes significantly increased the RH activity in the K12 derivatives (Figure 1D). RH purified from WQF8RH2 and MQF8RH2 displayed a specific activity of 0.03 U mg^–1^ and 0.07 U mg^–1^ (Figure 1D), which corresponds to a 1.5 and 3.5-fold increase, respectively, compared to the parental strains, which did not express the *C. necator hyp* genes. Remarkably, the strains WQF8RH3 and MQF8RH3, which co-express the *hyp1* operon without *hypF1*, showed even higher RH activities with a 2.5- and 6-fold increase, respectively (Figure 1D). These data highlight the positive effect of the co-expressed *C. necator* maturase genes on the catalytic activity of RH and underscore the previously observed requirement of the *hypF* gene of *E. coli* for RH maturation under anaerobic conditions (Lenz et al., 2007). Furthermore, *E. coli* MQF8RH3 was found to be most suitable for production of active RH as it showed the highest specific RH activity among the strains discussed so far. However, despite co-expression of the *C. necator hyp* genes, the activity of the heterologously produced RH remained very low.

### Addition of Nickel Improves the RH Activity in *E. coli*

**FIGURE 2:**
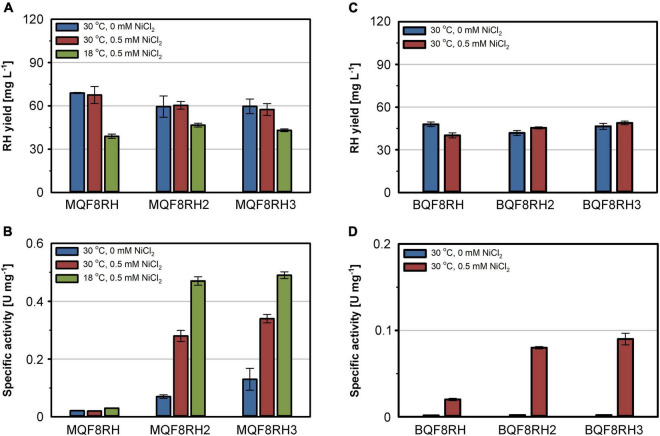
Effect of NiCl_2_ addition and cultivation temperature on RH yield and activity. Strains *E. coli* MQF8RH, MQF8RH2, MQF8RH3, BQF8RH, BQF8RH2 and BQF8RH3 were cultivated in 125 mL PreSens flask with 50 mL boosted EnPresso B medium under O_2_-limited conditions. After induction, the temperature was either maintained at 30°C or shifted to 18°C. NiCl_2_ (0.5 mM) was supplemented at the time point of the induction of RH gene expression as indicated. RH protein was purified by affinity chromatography **(A,C)** and specific activities measured from the purified samples **(B,D)**.

The comparatively low activity of the heterologously produced RH might result from an insufficient supply of nickel by the EnPresso B medium used for strain cultivation. Similar to LB, EnPresso B medium is a rich medium that most likely has a chelating effect on metal ions, thereby reducing their bioavailability (Rathnayake et al., 2013). To test this hypothesis, we added 500 μM nickel to the medium and cultivated the *E. coli* strains MQF8RH, MQF8RH2 and MQF8RH3 under microaerobic conditions. NiCl_2_ supplementation did neither affect the bacterial growth nor the yield of the purified RH (Figure 2A), excluding toxic effects of the additional nickel. In contrast, the extra nickel resulted in a significant increase in RH activity when the *C. necator hyp* machinery was co-expressed (Figure 2B). In fact, we observed an increase from 0.07 U mg^–1^ to 0.28 U mg^–1^ for the RH isolated from MQF8RH2 and from 0.13 U mg^–1^ to 0.34 U mg^–1^ for the RH isolated from strain MQF8RH3 cultivated at 30°C (Figure 2B). An even higher activity of 0.5 U mg^–1^ was obtained when the same strains were cultured at 18°C for an extended time period of 66 h after IPTG induction (see section “Materials and Methods”), although the lower temperature decreased the volumetric RH yield (Figure 2A). Remarkably, the RH proteins isolated from strains MQF8RH2 and MQF8RH3 cultivated at 18°C had similar specific activities, suggesting that the presence of the *C. necator hypF1* gene no longer impacted enzyme maturation (Figures 1D, 2B). The lower cultivation temperature is accompanied by an increased oxygen concentration, which could be the reason for the acquired functionality of HypF1. In conclusion, lowering the cultivation temperature and nickel supplementation markedly improved the specific activity of RH isolated from *E. coli* MC4100.

*Escherichia coli* BL21 Gold is known to be deficient in nickel uptake (Pinske et al., 2011). We thus tested whether the addition of NiCl_2_ improves the activity of the RH purified from strains BQF8RH, BQF8RH2, and BQF8RH3 cultivated at 30°C. Indeed, we detected a low activity of 0.09 U mg^–1^ for the RH purified from strain BQF8RH3 (Figure 2D). Thus, nickel availability appears to be the major impediment to [NiFe] cofactor assembly in *E. coli* BL21 Gold. However, the RH activity was approximately fourfold lower than that of the K-12 strain MQF8RH3 cultivated under the same conditions.

### Heterologously Produced RH Contains the Native NiFe (CO)(CN)_2_ Cofactor

**FIGURE 3:**
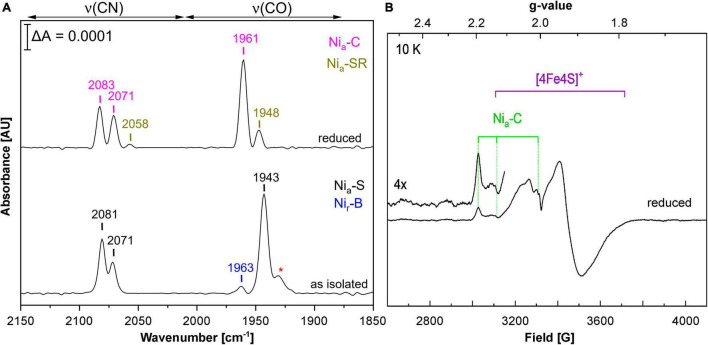
Spectroscopic characterization of RH heterologously produced in *E. coli.* Spectroscopic data were recorded on RH purified from MQF8RH2 (30°C/0.5 mM NiCl_2_). **(A)** IR spectra of as-isolated and H_2_-reduced RH. The signals indicate the presence of CO and CN ligands of the NiFe(CN)_2_CO active site. Bands are labeled according to the different redox states of the RH (Ash et al., 2015; Roncaroli et al., 2015; Caserta et al., 2020), i.e., Ni_a_-S (2,081, 2,071, 1,943 cm^–1^), Ni_r_-B (2,097, 2,089, 1,963 cm^–1^), Ni_a_-SR (2,073, 2,058, 1,948 cm^–1^), and Ni_a_-C (2,083, 2,071, 1,961 cm^–1^). The red asterisk most likely indicates traces of Ni_*r*_-S species (1,931 cm^–1^). **(B)** EPR spectrum of sodium dithionite (Na_2_S_2_O_4_)-reduced RH. The spectrum was recorded at 10 K and a microwave power of 1 mW.

Using *E. coli* MC4100 as host, we obtained RH activities that were about half of those reported for the first RH preparations from *C. necator* (Bernhard et al., 2001). This prompted us to spectroscopically investigate the cofactor content of purified RH from *E. coli* strain MQF8RH3, which was cultivated under O_2_-limiting conditions at 30°C and elevated nickel concentrations. By infrared spectroscopy, we monitored the CO and CN stretching vibrations of the CO and CN^–^ ligands of the NiFe(CN)_2_(CO) cofactor, which appear in a spectral region, in which no other protein-specific vibrations occur (De Lacey et al., 2007; Lubitz et al., 2014). The IR spectrum of the as-isolated, oxidized RH protein was dominated by the typical bands of the so-called Ni_a_-S state, characterized by a CO stretching band at 1,943 cm^–1^ and two cyanide stretchings at 2,071 and 2,081 cm^–1^. The IR signal amplitudes suggested a cofactor occupation of 5–10%. Treatment with H_2_ gas resulted in reduced RH, whose IR spectrum is dominated by the Ni_a_-C state with a CO stretching band at 1,961 cm^–1^ and two CN stretchings at 2,071 and 2,083 cm^–1^ (Figure 3A; Bernhard et al., 2001; Buhrke et al., 2005b; Roncaroli et al., 2015; Caserta et al., 2020). EPR spectroscopy, which allows the detection of paramagnetic states, confirmed the presence of signals related to the Ni_a_-C-state (g_x_ = 2.19, g_y_ = 2.13, g_z_ = 2.01) as well as [4Fe-4S]^1+^ cluster(s) signals in reduced RH treated with an excess of sodium dithionite (Figure 3B), which is in line with previous results obtained from RH isolated from *C. necator* (Roncaroli et al., 2015). These results demonstrate that the RH isolated from *E. coli* contains a canonical NiFe(CN)_2_(CO) cofactor that shares the same spectroscopic properties as the native RH isolated from *C. necator*.

### Optimal Metal Ion Supplementation for RH Activity in *E. coli*

**FIGURE 4:**
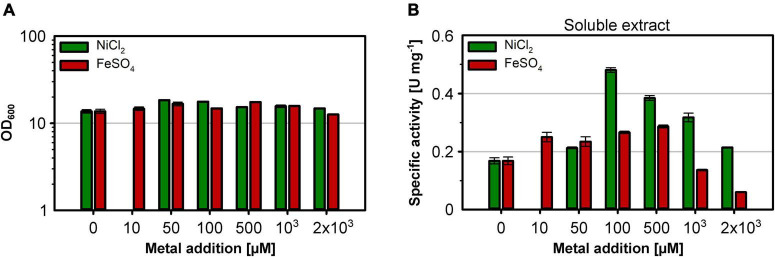
Effect of the Ni and Fe concentration on cell growth and RH activity. *E. coli* strain MQF8RH3 was cultivated in 24-deepwell plates with 8 mL boosted EnPresso medium at 30°C under O_2_-limited conditions. NiCl_2_ or FeSO_4_ were supplemented using the indicated concentrations varying from 0 to 2.0 mM. Cells were harvested 24 h after induction with 50 μM IPTG. **(A)** Optical density of the cell culture just before harvesting. **(B)** Soluble extracts were obtained by sonication and RH activity was measured. The specific RH activity was calculated based on the RH concentrations determined in the crude extracts by Western blotting (Supplementary Figure 4).

**FIGURE 5:**
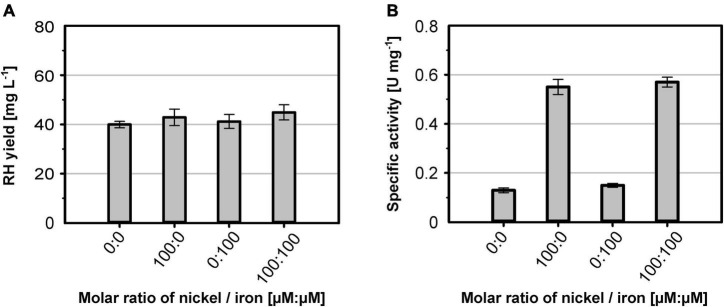
Metal effect on soluble RH yield and activity. *E. coli* strain MQF8RH3 was cultivated in 125 mL PreSens flasks with 50 mL boosted EnPresso B medium at 18°C. The medium was supplemented with different molar ratios of NiCl_2_ and FeSO_4_ as indicated. **(A)** RH protein was purified by affinity chromatography, and the yield was quantified. **(B)** Specific activities measured from the purified RH samples.

The nickel supplementation experiments described above were performed with a nickel concentration of 500 μM. To determine the optimal metal content in the growth medium, we devised a screening experiment with *E. coli* MQF8RH3 cultivated in 24-deepwell plates filled with EnPresso B medium containing different concentrations of nickel and iron. The cells were harvested, and the RH activity in crude cell extracts was determined as described in material and methods. The addition of up to 2 mM of either NiCl_2_ or FeSO_4_ had no effect on cell growth, as the final OD did not vary significantly (Figure 4A). As expected, NiCl_2_ addition led to an increase of RH activity in the crude extracts. The highest activity of 0.5 U mg^–1^ was observed upon addition of 100 μM NiCl_2_, which corresponds to an approximately threefold increase compared to the sample without nickel addition (Figure 4B). Further increase in nickel concentration diminished the RH activity (Figure 4B). In contrast, the addition of up to 500 μM FeSO_4_ had only a negligible effect on the RH activity in the crude extract. A further increase even reduced the RH activity below 50% of the level achieved without iron addition. Thus, metal concentrations higher than 100 μM Ni and 500 μM Fe had a negative effect on RH activity and were avoided in subsequent experiments. Based on these screening experiments, we investigated the effect of nickel and iron on RH production and activity in larger medium volumes. *E. coli* MQF8RH3 was cultivated at 18°C in EnPresso B supplemented with different nickel:iron ratios [0:0, 100:0, 0:100, 100:100 (μM:μM)]. RH was purified and its activity was measured (Figure 5). The RH yield was the same under all conditions (Figure 5A). An activity of approx. 0.13 U mg^–1^ was observed without the addition of nickel or iron (Figure 5B), which is consistent with our data at 30°C (Figure 2B). The activity did not vary significantly upon addition of 100 μM FeSO_4_, indicating that sufficient iron is present in the EnPresso B medium. In contrast, the addition of 100 μM NiCl_2_ resulted in a fourfold increase of specific activity of 0.55 U mg^–1^, which was similar to the value measured in the presence of 100 μM NiCl_2_/100 μM FeSO_4_ (0.57 U mg^–1^, Figure 5B). Based on these results, we added 100 μM NiCl_2_ but not further iron source to the growth media.

### Improved RH Maturation by Co-production of HoxN and HypX From *C. necator*

The results described above clearly show a significant activity increase when the RH is isolated from O_2_-limited *E. coli* cultures. Aerobic cultivation, by contrast, led to higher protein yields (Figure 1A). Therefore, we aimed to improve RH activity under aerobic conditions. Nickel supply is limited particularly under aerobic conditions as the endogenous nickel uptake system in *E. coli* in FNR-dependent and functional only under anaerobic conditions (Wu et al., 1989). Thus, we considered the gene encoding the high-affinity nickel permease HoxN, which mediates nickel uptake in *C. necator* under aerobic conditions (Eberz et al., 1989; Eitinger and Friedrich, 1991; Wolfram et al., 1995). Consequently, we extended plasmid pQF12 by adding *hoxN* gene from *C. necator* resulting into plasmid pQF17 (*hypA1B1F1CDE-hoxN)* (Supplementary Figure 1).

**FIGURE 6:**
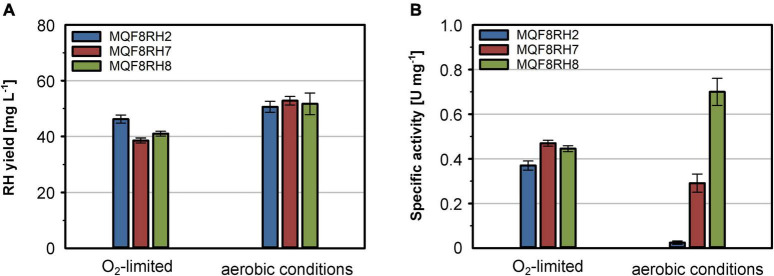
Effect of *hoxN1* and *hypX* genes on the production of active RH. *E. coli* strains MQF8RH2 (RH-Hyp1), MQF8RH7 (RH-Hyp1-HoxN1) and MQF8RH8 (RH-Hyp1-HoxN1-HypX) were cultivated at 18°C in 50 mL boosted EnPresso B medium supplemented with 0.1 mM NiCl_2_. After induction with 50 μM IPTG, RH production lasted for 48 h under either aerobic or O_2_-limited conditions. **(A)** RH protein was purified by affinity chromatography, and the yield was quantified. **(B)** Specific activities measured from the purified RH samples.

Additionally, we also considered that under aerobic conditions the formyltetrahydrofolate decarbonylase HypX from *C. necator* is responsible for the biosynthesis of the CO ligand of the NiFe(CN)_2_(CO) site (Bürstel et al., 2016; Schulz et al., 2020). Thus, we further extended plasmid pQF17 by implementing the *hypX* gene, yielding plasmid pQF18 (*hypA1B1F1CDEX-hoxN)*. The plasmids pQF17 and pQF18 were transferred to *E. coli* MQF8RH, resulting in strains MQF8RH7 and MQF8RH8, respectively. The new strains were grown in addition to strain MQF8RH2 in NiCl_2_-supplemented (100 μM) and boosted EnPresso B medium under both aerobic and O_2_-limited conditions. Cells were harvested after 48 h of IPTG induction, proteins purified and their specific activities quantified. While the presence of the new genes did not significantly affect the RH yield in the new strains (Figure 6A), the introduction of HoxN led to a slight increase of RH activity under O_2_-limited conditions and about 12-fold higher activity under aerobic conditions (Figure 6B). These data clearly indicate that HoxN is functional in nickel uptake in the presence of high O_2_ concentrations. The introduction of *hypX* did not improve the RH activity obtained from cells grown under O_2_-limiting conditions, in line with a so far elusive HypX-independent CO ligand synthesis (Soboh et al., 2013; Arlt et al., 2021). In contrast, the RH isolated from the aerobically grown strain MQF8RH8 showed a twofold higher specific activity (0.7 U mg^–1^) than the RH purified from strain MQF8RH7 (Figure 6B). These data, surprisingly, evidenced the successful implementation of HypX in *E. coli* strains able to synthesize the active site CO ligand under aerobic conditions. Taken together, these findings demonstrate that co-expression of HypX and HoxN in addition to the *C. necator hyp1* operon enables biosynthesis of active RH even under aerobic conditions. Additionally, we successfully increased the space-time yield of catalytically active RH by a factor of about 27 compared to strain MQF8RH2 cultivated under the same conditions (12.5 *vs.* 0.45 U (L d) ^–1^). The reported RH activity under aerobic conditions represents the highest activity for a heterologous produced RH so far.

### Production of Catalytically Active RH in *E. coli* BL21 Gold

**FIGURE 7:**
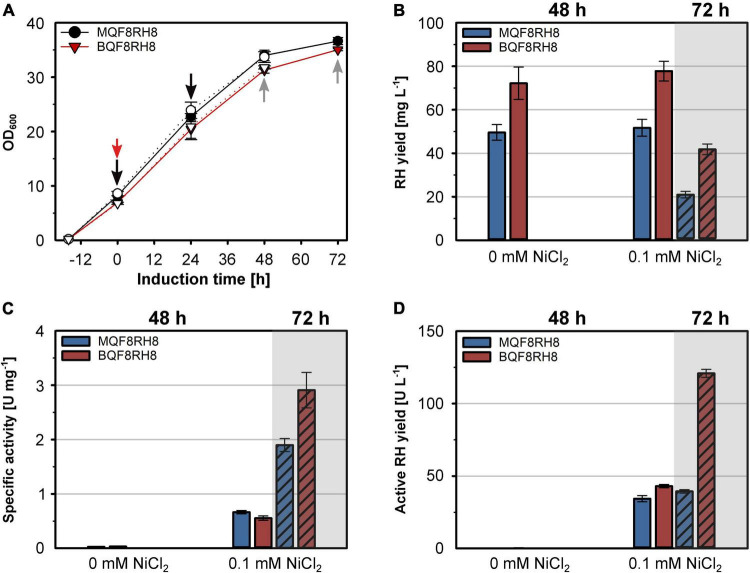
Production of active RH in aerated cultures of *E. coli* BL21 Gold. *E. coli* strains MQF8RH8 and BQF8RH8 were cultured in 50 mL boosted EnPresso B medium at 18°C under aerobic conditions. RH gene expression was induced with 50 μM IPTG and cells harvested 48 h or 72 h (hatched columns) after induction. NiCl_2_ was added at the time point of induction of gene expression as indicated. **(A)** Cell growth. The time point of induction is indicated by a red arrow, while the time points of booster addition and cell harvest are indicated by black and gray arrows, respectively. Open and filled symbols represent growth of *E. coli* with or without 0.1 mM NiCl_2_, respectively. **(B)** RH protein was purified by affinity chromatography, and the yield was quantified. **(B)** specific activities measured from the purified RH samples. **(D)** Volumetric yield of RH calculated from the results in panels **(B,C)**.

The successful heterologous production of active RH through co-expression of the *hyp* and *hoxN* genes under aerobic conditions prompted us to revisit strain BQF8RH that so far showed the highest RH yield, although inactive (Figure 1A). Hence, we transformed strain BQF8RH with plasmid pQF18 and investigated the RH production in the resulting strain BQF8RH8 using the MC4100 derivative MQF8RH8 for comparison. Both strains were cultivated under aerobic conditions with or without the addition of NiCl_2_. The added NiCl_2_ neither affected cell growth (Figure 7A) nor the RH yield of the two strains (Figure 7B). As expected, the *E. coli* BL21 derivative BQF8RH8 showed an about 50% higher RH yield compared to the K12 derivative MQF8RH8. However, the prolongation of the aerobic induction period from 48 h to 72 h resulted in a twofold decrease in RH yield for both strains (Figure 7B). Clearly, nickel supplementation is necessary for production of catalytically active RH (Figure 7C) when the strains are cultivated under aerobic conditions. After 48 h of induction, the specific activities of the RH preparations from both MQF8RH8 and BQF8RH8 ranged similarly at about 0.6 U mg^–1^. Despite the marked decrease in RH yield (Figure 7B), extension of the production period from 48 h to 72 h resulted in a threefold and fivefold increase in the specific RH activities of the samples isolated from strains MQF8RH8 and BQF8RH8 to 2 U mg^–1^ and even 3 U mg^–1^, respectively (Figure 7C). Accordingly, the specific RH activity of strain BQF8RH8 was almost as high as that of RH isolated from the native host *C. necator* (Caserta et al., 2020). Considering protein yield and specific activity, the yield of active RH was similar (40 U L^–1^ of culture) for both induction periods of MQF8RH8 and for BQF8RH8 after 48 h of induction (Figure 7D). Remarkably, after an induction time of 72 h, we obtained an unprecedented yield of active RH of 120 U L^–1^ (Figure 7D) for strain BQF8RH8.

## Discussion

Here, we present a major improvement in the heterologous hydrogenase production in *E. coli* exemplified by the purification of catalytically active RH from *C. necator*. We optimized important factors such as the cultivation mode (anaerobic/aerobic), Ni and Fe content in the medium, production temperature, and co-expression of specific maturation genes, thereby systematically increasing the H_2_-oxidizing activity. The RH activities as well as the protein yields obtained from all experiments conducted are summarized in Supplementary Table 3. Even though the RH is synthesized in an active form in its native host, *C. necator*, under aerobic conditions (Bernhard et al., 2001; Buhrke et al., 2005a; Ash et al., 2015), no active RH could be purified from aerobically grown *E. coli* strains without further genetic amendments. Under microaerobic/anaerobic growth conditions, however, little RH activity was observed, consistent with the fact that the hydrogenase maturation apparatus of *E. coli* is functional only under anaerobiosis (Messenger and Green, 2003; Forzi and Sawers, 2007; Lenz et al., 2007). Nevertheless, the very low activity suggests that the *E. coli* Hyp maturases are inefficient in outfitting the RH with the NiFe(CN)_2_CO cofactor. Indeed, the co-expression of the *hyp1* operon encoding the HypA1, B1, F1, C, D, and E proteins from *C. necator* further increased RH activity in *E. coli* (Figure 1). Although the Hyp proteins of *E. coli* and *C. necator* show an amino acid sequence identity of 18–45% (Wolf et al., 1998; Böck et al., 2006; Vignais and Billoud, 2007), this observation clearly demonstrates that they are not generally interchangeable with each other. The HypF protein plays an interesting role here. HypF1 from *C. necator* seems to be inactive under anaerobic conditions, and the *E. coli* HypF ortholog of *E. coli* takes over its function, consistent with a previous report (Lenz et al., 2007). Moreover, both HypF versions seem to compete with each other, as there is an increase in RH activity under anaerobic conditions in the absence of HypF1 of *C. necator*. The likely reason is that the inactive version of *C. necator* interferes with the interaction of the HypE protein with the *E. coli* HypF. Notably, the HypF1 of *C. necator* comprises only one of three domains present in canonical HypF proteins, such as that from *E. coli* (Wolf et al., 1998; Paschos et al., 2002). While canonical HypF proteins synthesize cyanide (CN) residue from carbamoyl phosphate and ATP, the truncated HypF likely requires carbamoyl adenylate for CN synthesis (Reissmann et al., 2003; Blokesch et al., 2004b). It is possible that carbamoyl adenylate is produced in sufficient quantities in living cells only under aerobic conditions. This assumption is supported by the higher RH activities obtained at lower temperature (Figure 2) which can be explained by a higher oxygenation of the medium compared to higher temperatures. Furthermore, lower temperatures might prevent misfolding of both the RH subunits and the maturases.

As nickel and iron are essential for RH activity, both metal ions must be provided in sufficient quantities. The availability of the metal ions depends both on their concentration in the medium, their bioavailability and the presence of specific cellular uptake systems. In *E. coli*, Fe^2+^ is predominantly transported across the membrane by the Feo system under microaerobic and anaerobic growth conditions, while Fe^3+^, occurring predominantly under aerobic conditions, is taken up by the Fec system (Braun and Hantke, 2011; Kosman, 2013; Lau et al., 2016). Thus, the versatile iron uptake systems in *E. coli* allow for sufficient intracellular availability and iron homeostasis under both aerobic and anaerobic conditions. Kim et al. (2010, 2011) reported that iron rather than nickel needs to be added for the production of active *E. coli* hydrogenase 1 and *Hydrogenovibrio marinus* [NiFe]-hydrogenase in *E. coli* in a modified minimal medium. In the case of EnPresso B, the addition of iron had a negligible effect on the RH activity (Figures 4, 5), indicating that this medium already contains sufficient iron. In contrast to iron, nickel is much less abundant and might be limiting in case of the overproduction of nickel-containing proteins such as [NiFe]-hydrogenase. In *E. coli*, nickel is taken up *via* the specific Nik system, which is synthesized only under anaerobic conditions. In Nik*-*deficient strains, such as *E. coli* BL21, nickel can be taken up by the magnesium transport system, which, however, has much lower specificity for nickel than the Nik transporter (Eitinger and Mandrand-Berthelot, 2000). In line with previous results that a high concentration (0.5 mM) of NiCl_2_ added to the medium restores the activity of *E. coli* hydrogenases in strains with defects in nickel uptake (Waugh and Boxer, 1986; Wu et al., 1989), the activity of the RH improves significantly in all tested strains upon nickel addition (Figures 2–5). Interestingly, not only the Nik-deficient BL21 derivatives but also the MC4100 derivatives showed nickel dependence, indicating that the chelating properties of the medium limit the availability of nickel. The co-production of the high-affinity nickel permease HoxN from *C. necator* resulted in a substantial increase in RH activity both under anaerobic and aerobic growth conditions. Moreover, the presence of HoxN enables RH maturation even in the presence of molecular oxygen (Figure 6). Notably, the addition of up to 0.1 mM NiCl_2_ to our EnPresso B medium had no effect on cell growth, even in the presence of HoxN.

It has been previously shown that under aerobic conditions the availability of CO is limiting for maturation of the NiFe(CN)_2_CO cofactor of hydrogenase (Bürstel et al., 2011). To address this potential problem in case of the production of hydrogenase in aerobically grown *E. coli*, we co-expressed the auxiliary *hypX* gene from *C. necator*, which is known to encode a CoA-dependent formyl-tetrahydrofolate (THF) decarbonylase (Schulz et al., 2020). This strategy improved the RH activity by threefold compared to a strain producing the HoxN permease alone (Figure 6). Remarkably, co-expression of *hoxN* and *hypX* genes in aerobically grown cells resulted in RH activities that were above those measured for RH isolated from anaerobically grown cells (Figure 7). Here, prolonged cultivation time from 48 h to 72 h significantly improved the specific RH activity, suggesting that the active site formation seems to be clearly dependent on post-induction time. It is possible that the biosynthesis and incorporation of active site is a slow process, as it requires the involvement of multiple active maturases. This assumption is supported by the linear and continuous increase in specific RH activities with increasing induction time upon induction from 6 h to 72 h. Therefore, prolonging the induction time might serve as a further alternative strategy to maximize the cofactor occupancy in the active center and increase hydrogenase activity.

## Conclusion

Commercial use of [NiFe]-hydrogenases is limited by the difficulty of producing these biocatalysts in scalable quantities. With some important exceptions, [NiFe]-hydrogenases are usually isolated from their native hosts (Fan et al., 2020). This is due to the fact that these complex metalloenzymes require a sophisticated maturation machinery. With this study we demonstrated that it is possible to produce a non-native [NiFe]-hydrogenase recombinantly in *E. coli* with high yield and a catalytic activity that—most importantly—is equivalent to that of hydrogenase purified from the native host. We purified the O_2_-tolerant regulatory hydrogenase from *C. necator* from three *E. coli* derivatives with different genetic backgrounds under various growth conditions. Whereas Bernhard et al. and Caserta et al. obtained approx. 1 U L^–1^ (1.1 mg L^–1^ of RH with an activity of 1 U mg^–1^) (Bernhard et al., 2001) and 0.4 U L^–1^ (0.1 mg L^–1^, 4.5 U mg^–1^) (Caserta et al., 2020), respectively, of purified RH from the native host, *C. necator*, our heterologous production process achieved 120 U L^–1^ (40 mg L^–1^ with 3 U mg^–1^). This corresponds to an 100–300-fold higher yield of catalytically active RH. The highest yield was obtained from aerobically cultivated of *E. coli*, which significantly shortens the process time compared to the production in *C. necator*. Through strain improvement, i.e., the co-expression of specific maturation genes, and process optimization, we were even able to produce a catalytically active metalloprotein in the metabolically deficient *E. coli* BL21. Through optimally controlled expression of the required genes and optimized production conditions, the combinational strategy developed in this work offers a clear advantage over the native producer. Our strategy provides a useful roadmap for biotechnologically relevant production of [NiFe]-hydrogenases and can be applied in scale-up studies to achieve commercial feasibility of complex, difficult-to-produce metalloenzymes.

## Data Availability Statement

The raw data supporting the conclusions of this article will be made available by the authors, without undue reservation.

## Author Contributions

MG, QF, and OL participated in experimental design and interpretation of the results. QF carried out all molecular biological and biochemical experiments, and prepared the original draft. GC, CL, and IZ performed the spectroscopic IR and EPR measurements and the data evaluation. GC, CL, IZ, PN, and OL analyzed the data with major contributions from QF and MG. MG, PN, and OL revised the manuscript. All authors read and approved the final manuscript.

## Conflict of Interest

PN is a shareholder of EnPresso GmbH. The remaining authors declare that the research was conducted in the absence of any commercial or financial relationships that could be construed as a potential conflict of interest.

## Publisher’s Note

All claims expressed in this article are solely those of the authors and do not necessarily represent those of their affiliated organizations, or those of the publisher, the editors and the reviewers. Any product that may be evaluated in this article, or claim that may be made by its manufacturer, is not guaranteed or endorsed by the publisher.
