# Supplementary material for: High-Yield Production of Catalytically Active Regulatory [NiFe]-Hydrogenase From Cupriavidus necator in Escherichia coli
Source: Front Microbiol. 2022 Apr 29;13:894375. doi: 10.3389/fmicb.2022.894375 (PMC9100943; doi:10.3389/fmicb.2022.894375)
Supplement: Supplementary file 1 [file Data_Sheet_1.PDF]

Supplementary Material

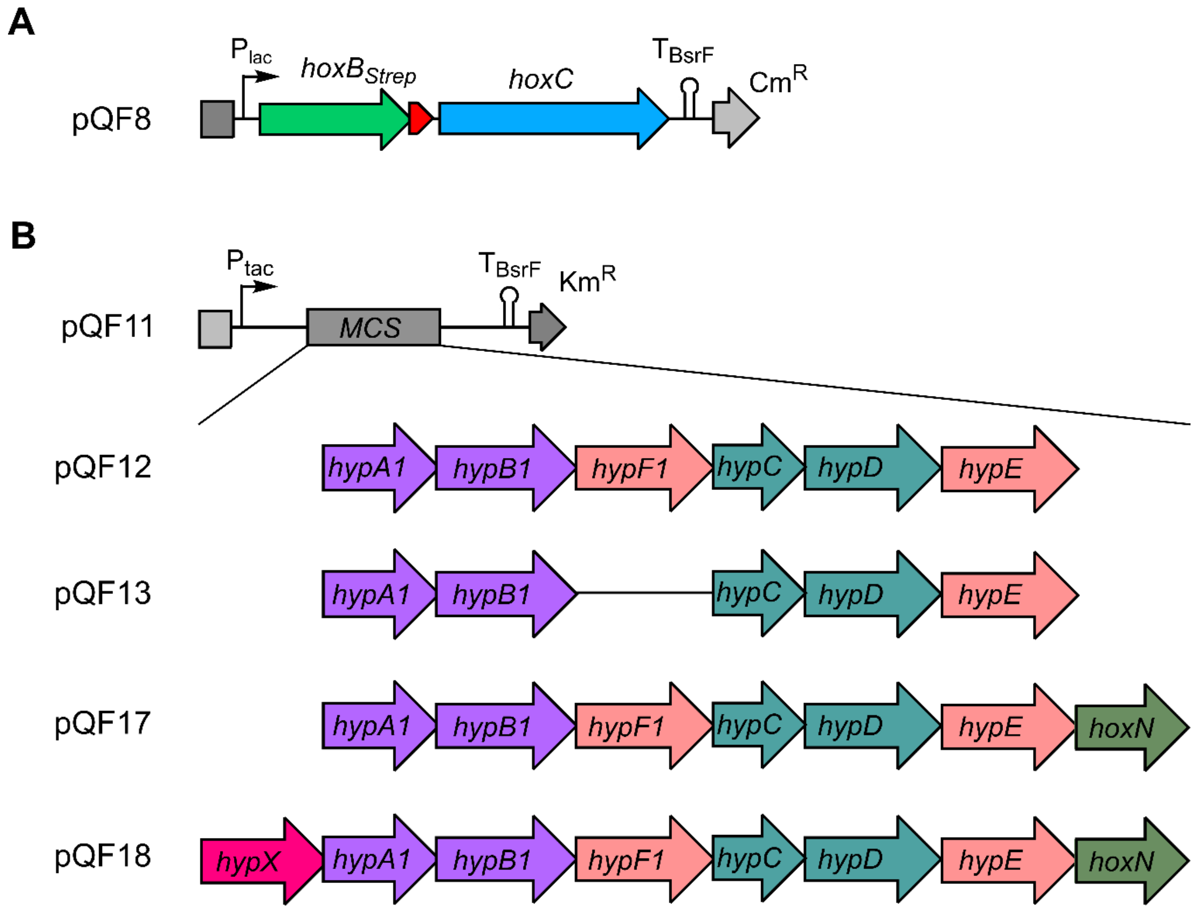

Figure S1. Schematic representation of *hox* and *hyp* expression cassettes.

(A) Organization of the expression cassette for the RH structural genes (*hoxB*C) on plasmid pQF8. **(B)** Organization of the gene cassettes for expression of the maturation proteins *hypA1B1F1CDEX* and *hoxN1* on pQFxx plasmids. Transcription start sites and transcription terminators are indicated by kinked arrows and stemloops, respectively. Cm^R: chloramphenicol resistance gene; Km^R kanamycin resistance gene.

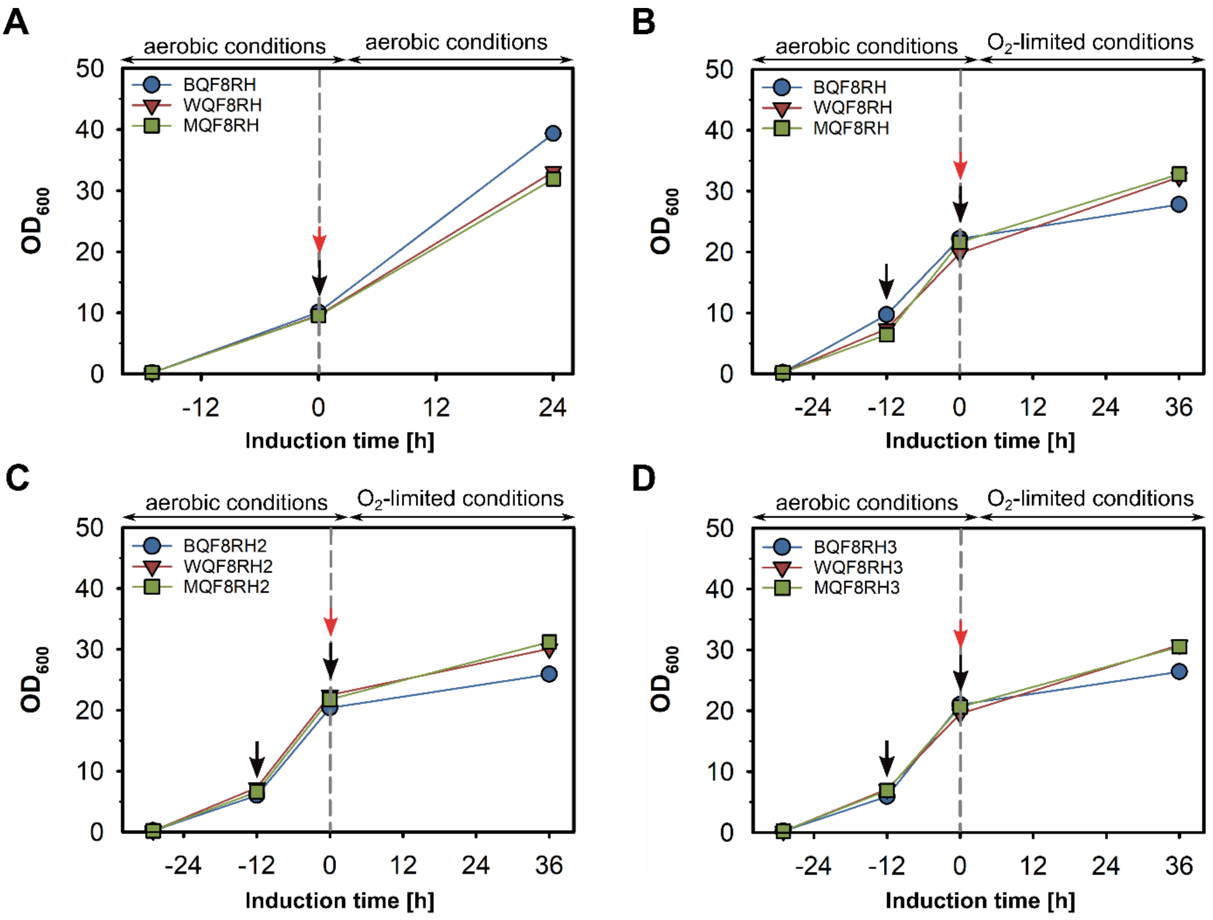

Figure S2. Cell growth of strains from Figure 1.

E. coli strains BQF8RH, WQF8RH and MQF8RH (derivatives of *E. coli* strains BL21 Gold, W3110 and MC4100, respectively) each carrying plasmid pQF8 encoding the RH structural subunits were cultivated in 50 mL EnPresso B medium as described in Materials and Methods. For aerobic production (**A**), cultivation was continued in 250-mL Ultra Yield flasks (20% V/V) shaken at 250 rpm, whereas for O_2-limited production (**B**) cultures were transferred to 125-mL PreSens flasks (40% V/V) after induction with 50 µM IPTG and the DO adjusted to about 0% by manually decreasing the shaking speed. The induction point is indicated by a red arrow. Booster and 75 µl reagent A (4.5 U L^{-1}) were added at the induction point. To ensure high cell densities, a 1st dose of booster was already added 12 h before induction. The booster addition is indicated by a black arrow. (**C**) Cultivation (as in B) of strains BQF8RH2, MQF8RH2 and WQF8RH2 each expressing the *hyp1* operon in addition to the RH structural genes. (**D**) Cultivation (as in B) of strains BQF8RH3, MQF8RH3 and WQF8RH3 each expressing the modified *hyp1*(ΔF1) operon in addition to the RH structural genes.

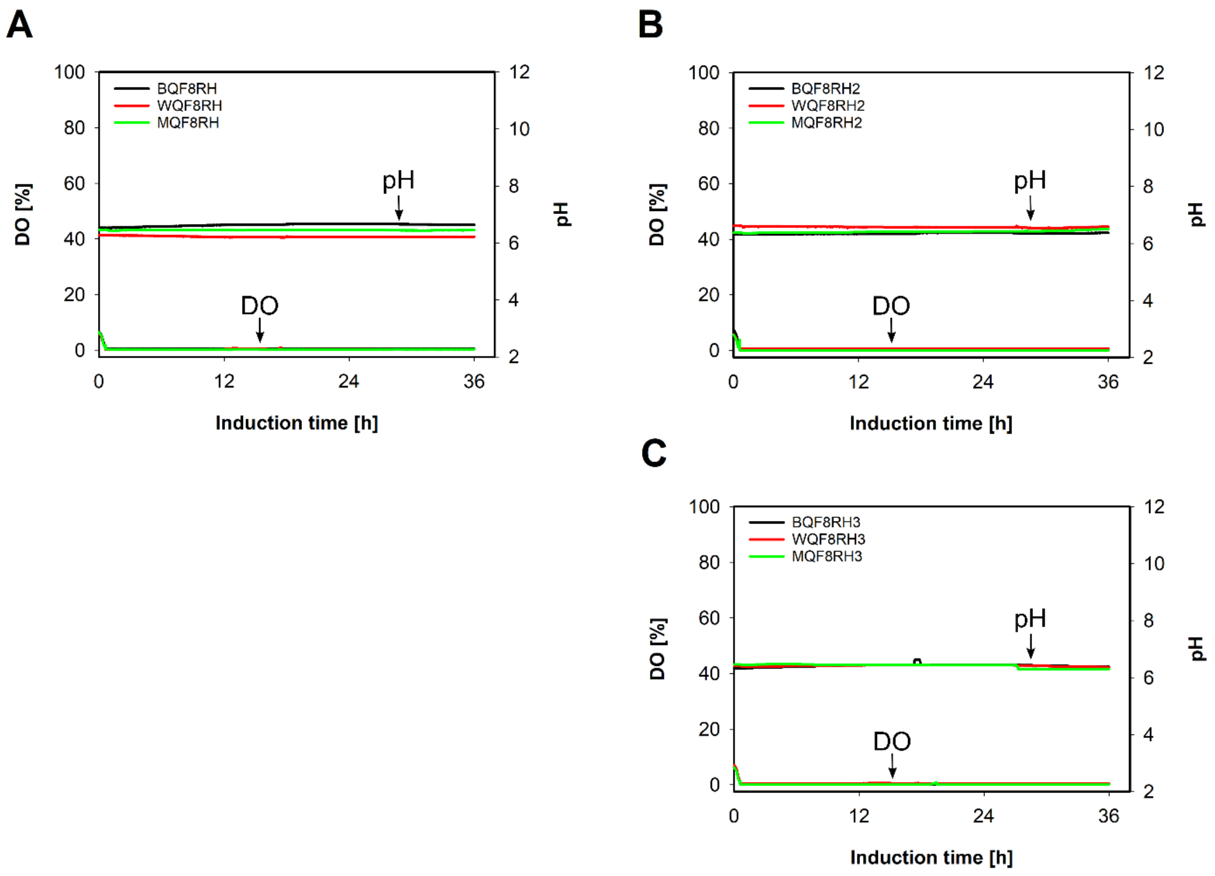

Figure S3. DO and pH values of strains from Figure 1 under O_2-limited culture conditions.

E. coli strains BQF8RH, WQF8RH and MQF8RH (derivatives of *E. coli* strains BL21 Gold, W3110 and MC4100, respectively) each carrying plasmid pQF8 encoding the RH structural subunits were cultivated in 50 mL EnPresso B medium as described in Materials and Methods. Subsequently, strains BQF8RH, MQF8RH and WQF8RH were transformed with either plasmid pQF12 (encoding the entire *hyp1* operon) or plasmid pQF13 (encoding the modified *hyp1(ΔF1)* operon lacking *hypF1*) yielding strains BQF8RH2, WQF8RH2 or MQF8RH2 and BQF8RH3, WQF8RH3 or MQF8RH3, respectively. For aerobic production, cultivation was performed in 250-mL Ultra Yield flasks (20% V/V) shaken at 250 rpm, O_2-limited production was performed in 125-mL PreSens flasks (40% V/V) adjusted to a DO near 0% by manually decreasing the shaking speed. RH protein was purified by affinity chromatography (**A, C**) and specific activities measured from the purified samples (**B, D**) as described recently (Fan et al., 2021).

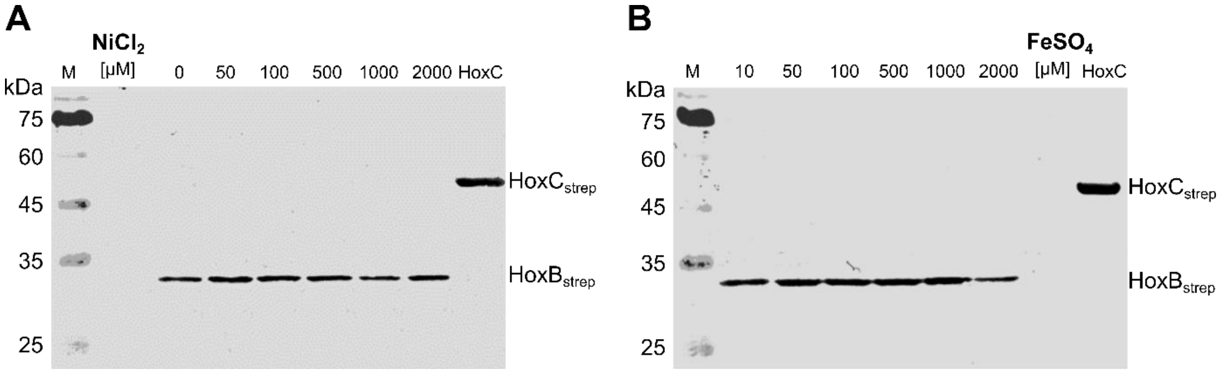

Figure S4. Western Blot analysis.

The pellets from 8 mL culture broth were resuspended with 2.5 mL 50 mM KH_2PO_4/K_2HPO_4 buffer pH 7.0 supplemented 1 mM PMSF and 1 mg mL-1 lysozyme and the cells were disrupted with ultrasonication for 3 min on ice (30 s on/off, 7 mm sonotrode diameter, 40 % amptitude) followed by centrifugation (16,000 xg, 4 °C, 30 min). 20 µL soluble protein extract was mixed with 2xSDS sample buffer and heated at 95 °C for 10 min. For each sample, 15 µL was used on 12% SDS-PAA gels followed by Western blotting. HoxB from RH was detected on the WB using anti-Strep-tag antibody. 1.5 µg purified $HoxC_{strep}$ was used as control on the SDS-PAA gels.

Table S1. Strains used in this study

E. coli strain	Genotyp	Reference
TG1	*E. coli K-12 glnV44 thi-1 Δ(lac-proAB) Δ(mcrB-hsdSM)5(r_{K}–m_{K}–)* *F' [traD36 proAB^{+} lacI^{q} lacZΔM15]*	Baer *et al.*, 1984
BL21 Gold	*E. coli B F$^{–}$ompT hsdS(r_{B}– m_{B}–) dcm^{+} Tet^{R} gal endA Hte*	Agilent, Waldbronn
W3110	*E. coli K-12 F- λ- rph-1 INV(rrnD, rrnE)*	Bachman et al., 1972
MC4100	*E. coli K-12 F- [araD139]$_{B/r}$ Δ(argF-lac)169 e14^{-} flhD5301 relA1 deoC1 Δ(fruK-yeiR)725(fruA25) rpsL150(strR) rbsR22 Δ(fimB-fimE)632(::IS1) λ^{-}*	Casadaban et al. 1979
BQF8RH	as BL21 Gold with plasmid pQF8	Fan et al., 2021
BQF8RH2	as BL21 Gold with plasmids pQF8 and pQF12	This work
BQF8RH3	as BL21 Gold with plasmids pQF8 and pQF13	This work
BQF8RH5	as BL21 Gold with plasmids pQF8 and pQF18	This work
MQF8RH	as MC4100 with plasmid pQF8	This work
MQF8RH2	as MC4100 with plasmids pQF8 and pQF12	This work
MQF8RH3	as MC4100 with plasmids pQF8 and pQF13	This work
MQF8RH4	as MC4100 with plasmids pQF8 and pQF17	This work
MQF8RH5	as MC4100 with plasmids pQF8 and pQF18	This work
WQF8RH	as W3110 with plasmid pQF8	This work
WQF8RH2	as W3110 with plasmids pQF8 and pQF12	This work
WQF8RH3	as W3110 with plasmids pQF8 and pQF13	This work

Table S2. Plasmids used in this study

Plasmid	Description	Reference
pRH-Hyp	pCM62 with *hoxBC* and *hyp1* operon (*hypA1B1F1CDE*), Tet^R	Lenz et al., 2007
pRH-Hyp(ΔF1)	pCM62 with *hoxBC* and *hyp1*(*ΔF1*) operon (*hypA1B1CDE*), Tet^R	Lenz et al., 2007
pCH231	pBluescript KS^+ with *hoxN*, Amp^R	Eitinger & Friedrich 1991
pGE771	pEDY309 with *hoxFUYHWIhypA2B2F2CDEXhoxA*, Tet^R	Lauterbach & Lenz, 2013
pGK14	*E. coli, S. thermophilus* shuttle vector Ery^R	Brantl, 1994
pGK16	pGK14 derivative, MCS, exchange of Ery^R to Km^R	Gimpel, unpublished
pGW2	*E. coli* expression vector, P_{tac}, MCS, T_{BsrF}, Amp^R	Schollmeyer, 2020
pQF8	*E. coli* cloning vector, P_{lac}, *hoxB*$_{Strep}$*C*; Cm^R	Fan et al., 2021
pQF11	as pGK16 with P_{tac}, MCS, T_{BsrF} from pGW2, Km^R	This work
pQF12	as pQF11 with *hyp1* operon from pRH-Hyp, Km^R	This work
pQF13	as pQF11 with *hyp1*(*ΔF1*) operon from pRH-Hyp(ΔF1), Km^R	This work
pQF17	as pQF12 with *hoxN* from pCH231, Km^R	This work
pQF18	as pQF17 with *hypX* from pGE771, Km^R	This work

Table S3. RH yields and activities obtained in this study.

Strain	Maturation genes	Culture conditions: Aeration	Temp.	Metal supplementation	Induction [h]	RH yield [mg/L]	Specific activity [U/mg]	Activity yield [U/L]	Productivity [U/(L*d)]
BQF8RH	-	aerobic	30°C	-	24	**124**	<0.001	ND	ND
BQF8RH	-	O_2-limited	30°C	-	36	49	<0.001	ND	ND
BQF8RH	-	O_2-limited	30°C	0.5 mM $NiCl_2$	36	39	0.02	0.933	0.41
BQF8RH2	*hyp1*	O_2-limited	30°C	-	36	43	<0.002	ND	ND
BQF8RH2	*hyp1*	O_2-limited	30°C	0.5 mM $NiCl_2$	36	45	0.08	3.737	1.66
BQF8RH3	*hyp1(ΔF1)*	O_2-limited	30°C	-	36	45	<0.001	ND	ND
BQF8RH3	*hyp1(ΔF1)*	O_2-limited	30°C	0.5 mM $NiCl_2$	36	50	0.09	4.711	2.09
BQF8RH8	*hyp1-hoxN-hypX*	aerobic	18°C	-	48	72	0.01	0.38	0.14
BQF8RH8	*hyp1-hoxN-hypX*	aerobic	18°C	0.1 mM $NiCl_2$	48	78	0.55	43.06	15.66
BQF8RH8	*hyp1-hoxN-hypX*	aerobic	18°C	0.1 mM $NiCl_2$	72	41	**2.92**	**120.59**	**32.16**
MQF8RH	-	aerobic	30°C	-	24	80	0.00	0.13	0.08
MQF8RH	-	O_2-limited	30°C	-	36	69	0.02	1.45	0.64
MQF8RH	-	O_2-limited	30°C	0.5 mM $NiCl_2$	36	68	0.02	1.47	0.65
MQF8RH	-	O_2-limited	18°C	0.5 mM $NiCl_2$	66	39	0.03	1.15	0.33
MQF8RH2	*hyp1*	O_2-limited	30°C	-	36	60	0.07	4.22	1.87
MQF8RH2	*hyp1*	O_2-limited	30°C	0.5 mM $NiCl_2$	36	60	0.28	16.83	7.48
MQF8RH2	*hyp1*	aerobic	18°C	0.1 mM $NiCl_2$	48	51	0.02	1.24	0.45
MQF8RH2	*hyp1*	O_2-limited	18°C	0.5 mM $NiCl_2$	66	47	0.47	21.91	6.26
MQF8RH2	*hyp1*	O_2-limited	18°C	0.1 mM $NiCl_2$	72	54	0.48	25.88	6.90
MQF8RH3	*hyp1(ΔF1)*	O_2-limited	30°C	-	36	60	0.13	7.69	3.42
MQF8RH3	*hyp1(ΔF1)*	O_2-limited	30°C	0.5 mM $NiCl_2$	36	57	0.34	19.49	8.66
MQF8RH3	*hyp1(ΔF1)*	O_2-limited	18°C	0.5 mM $NiCl_2$	66	43	0.49	21.21	6.06
MQF8RH3	*hyp1(ΔF1)*	O_2-limited	18°C	-	72	39	0.13	5.07	1.35
MQF8RH3	*hyp1(ΔF1)*	O_2-limited	18°C	0.1 mM $NiCl_2$	72	41	0.55	22.28	5.94
MQF8RH3	*hyp1(ΔF1)*	O_2-limited	18°C	0.1 mM $FeSO_4$	72	43	0.15	6.48	1.73
MQF8RH3	*hyp1(ΔF1)*	O_2-limited	18°C	0.1 mM $NiCl_2$. 0.1 mM FeSO4	72	43	0.57	24.34	6.49
MQF8RH7	*hyp1-hoxN*	aerobic	18°C	0.1 mM $NiCl_2$	48	53	0.29	15.38	5.59
MQF8RH7	*hyp1-hoxN*	O_2-limited	18°C	0.1 mM $NiCl_2$	48	39	0.47	18.20	6.62
MQF8RH7	*hyp1-hoxN*	O_2-limited	18°C	0.1 mM $NiCl_2$	72	51	0.84	42.96	11.45
MQF8RH8	*hyp1-hoxN-hypX*	aerobic	18°C	0.1 mM $NiCl_2$	48	52	0.66	34.34	12.49
MQF8RH8	*hyp1-hoxN-hypX*	aerobic	18°C	-	48	50	0.00	0.19	0.07
MQF8RH8	*hyp1-hoxN-hypX*	aerobic	18°C	0.1 mM $NiCl_2$	72	21	1.91	39.16	10.44
MQF8RH8	*hyp1-hoxN-hypX*	O_2-limited	18°C	0.1 mM $NiCl_2$	48	41	0.45	18.29	6.65
WQF8RH	-	aerobic	30°C	-	24	92	0.00	0.09	0.05
WQF8RH	-	O_2-limited	30°C	-	36	69	0.02	1.24	0.55
WQF8RH2	*hyp1*	O_2-limited	30°C	-	36	65	0.03	1.88	0.84
WQF8RH3	*hyp1(ΔF1)*	O_2-limited	30°C	-	36	70	0.05	3.28	1.46
